# Dual function of the nuclear export signal of the Borna disease virus nucleoprotein in nuclear export activity and binding to viral phosphoprotein

**DOI:** 10.1186/s12985-017-0793-6

**Published:** 2017-07-11

**Authors:** Mako Yanai, Madoka Sakai, Akiko Makino, Keizo Tomonaga

**Affiliations:** 1 Laboratory of RNA virus, Institute for Frontier Life and Medical Sciences, Kyoto University, 53 Kawahara-cho, Shogoin, Sakyo-ku, Kyoto, 606-8507 Japan; 2 Department of Mammalian Regulatory Network, Graduate School of Biostudies, Kyoto University, Kyoto, Japan; 3 Department of Molecular Viruses, Graduate School of Medicine, Kyoto University, Kyoto, Japan

**Keywords:** Borna disease virus, Nucleocytoplasmic shuttling, Nuclear export signal, Nucleoprotein-phosphoprotein interaction, Polymerase activity

## Abstract

**Background:**

Borna disease virus (BoDV), which has a negative-sense, single-stranded RNA genome, causes persistent infection in the cell nucleus. The nuclear export signal (NES) of the viral nucleoprotein (N) consisting of leucine at positions 128 and 131 and isoleucine at positions 133 and 136 overlaps with one of two predicted binding sites for the viral phosphoprotein (P). A previous study demonstrated that higher expression of BoDV-P inhibits nuclear export of N; however, the function of N NES in the interaction with P remains unclear. We examined the subcellular localization, viral polymerase activity, and P-binding ability of BoDV-N NES mutants. We also characterized a recombinant BoDV (rBoDV) harboring an NES mutation of N.

**Results:**

BoDV-N with four alanine-substitutions in the leucine and isoleucine residues of the NES impaired its cytoplasmic localization and abolished polymerase activity and P-binding ability. Although an alanine-substitution at position 131 markedly enhanced viral polymerase activity as determined by a minigenome assay, rBoDV harboring this mutation showed expression of viral RNAs and proteins relative to that of wild-type rBoDV.

**Conclusions:**

Our results demonstrate that BoDV-N NES has a dual function in BoDV replication, i.e., nuclear export of N and an interaction with P, affecting viral polymerase activity in the nucleus.

## Background

Borna disease virus (BoDV) is an enveloped virus with a non-segmented, negative-strand RNA genome [1] and establishes persistent infection in the host cell nucleus [2]. The BoDV ribonucleoprotein (vRNP) consists of viral genome RNA (vRNA), nucleoprotein (N), large protein (L), phosphoprotein (P), and matrix protein (M). BoDV-N forms an N-vRNA complex through viral RNA encapsidation [3, 4]. BoDV-L, an RNA-dependent RNA polymerase, acts with P for viral transcription and replication [5]. BoDV-P functions as a hub for vRNP formation by binding to N and L [6–8]. Although BoDV-M has no effect on viral polymerase activity, it may play a role in the transport of vRNP by binding to viral RNA and P [9, 10]. When BoDV vRNP is released into the cytoplasm after entry, vRNP is transported into the cell nucleus where BoDV transcription and replication occur [2, 11]. Newly synthesized N, L, and P are also transported into the nucleus and form vRNP. The progeny vRNP is enveloped with the host plasma membrane and buds as a progeny virion [12]. In this manner, BoDV vRNP shuttles between the cytoplasm and nucleus during its replication. The nuclear localization signal (NLS) in N, L, and P and nuclear export signal (NES) in N and P play important roles in nucleocytoplasmic trafficking of BoDV vRNP [13–16].

**Fig. 1:**
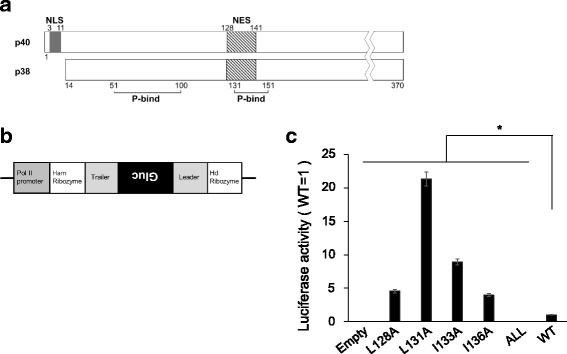
Minigenome assay of BoDV-N NES mutants. Schematic diagram of BoDV-N (**a**) and BoDV minigenome (**b**). **c**. Minigenome assay using BoDV-N NES mutants. 293 LTV cells were transfected with pC-BoDV-MG-Gluc, pC-BoDV-N, pC-BoDV-L, and pCXN2-P. At 48 h post-transfection, luciferase activity was measured. The experiments were performed independently three times. The data were analyzed with Student’s *t*-test. Bars show mean ± standard deviation (SD) (*; *p* value <0.05)

BoDV-N is found as two isoforms, p40 (full-length) and p38, in infected cells. p38 is translated from the second start codon in the N open reading frame and thus contains no NLS at its N-terminus (Fig. 1a) [17]. When p40 or p38 of BoDV-N is expressed in cells alone, p40 and p38 are observed in the nucleus and cytoplasm, respectively [13, 17]. In contrast, co-expression of p40 and p38 leads to their localization in both the nucleus and cytoplasm [13]. The BoDV-N NES (at the position of 128–141) overlaps with one of the BoDV-P binding sites (131–158) [18] (Fig. 1a), and NES activity is controlled by the interaction with P [13]. BoDV-N nucleocytoplasmic shuttling is regulated by the expression levels of its two isoforms and P. Previous studies evaluated the nuclear export activity of BoDV-N by using N mutants or the NES fused with GFP, but the role of the BoDV-N NES in viral replication remains unclear. Here, to determine whether BoDV-N NES is directly involved in viral replication, we analyzed its interaction with BoDV-P and viral polymerase activity by using N NES mutants and characterized rBoDV containing an NES mutation.

## Results and discussion

Nuclear export of BoDV-N occurs via a CRM1-dependent pathway [13]. The canonical NES of BoDV-N consists of two leucine (at positions 128 and 131) and two isoleucine (133 and 136) residues (Table 1). We introduced alanine-substitutions at each or all of the four amino acid residues of the NES (Table 1) and generated expression plasmids of BoDV-N NES mutants using pCAGGS [19].

**Table 1:** BoDV N mutants used in this study

Construct	NES sequence								
pC-BoDV-N (WT)	L	T	E	L	E	I	S	S	I
pC-BoDV-N-L128A (L128A)	A	-	-	-	-	-	-	-	-
pC-BoDV-N-L131A (L131A)	-	-	-	A	-	-	-	-	-
pC-BoDV-N-I133A (I133A)	-	-	-	-	-	A	-	-	-
pC-BoDV-N-I136A (I136A)	-	-	-	-	-	-	-	-	A
pC-BoDV-N-NES-A (ALL)	A	-	-	A	-	A	-	-	A

To evaluate viral polymerase activity, we used a modified minigenome assay as previously reported [20]. Briefly, the BoDV minigenome RNA was transcribed by Pol II from a cDNA coding the antisense open reading frame of Gaussia luciferase (Gluc), a secreted reporter gene, inserted between the 5′ trailer and 3′ leader sequences of BoDV (Fig. 1b). This construct was used as a template for transcription and replication. Next, 1 × 10^5^ 293 LTV cells (Cell Biolabs, Inc., San Diego, CA, USA) were transfected with 0.125 μg of BoDV minigenome (pC-BoDV-MG-Gluc), BoDV-N and L expression plasmids (pC-BoDV-N and pC-BoDV-L), and 0.0125 μg of the P expression plasmid (pCXN2-P) [20] using Lipofecatmine® 2000 (Thermo Fisher Scientific, Waltham, MA, USA). At 48 h post-transfection, the culture supernatants were collected and luciferase activities were measured using the BioLux® Gaussia Luciferase Assay Kit (New England BioLabs, Ipswich, MA, USA). When a BoDV-N expression plasmid, in which all leucine and isoleucine residues in the NES were substituted with alanine, was transfected with BoDV minigenome, viral polymerase activity was completely lost (Fig. 1c), suggesting that the NES sequence is essential for viral polymerase activity. Surprisingly, a single alanine-substitution at each of the four residues of the NES, particularly the L131A mutant, significantly increased viral polymerase activity compared to wild-type BoDV-N (Fig. 1c).

**Fig. 2:**
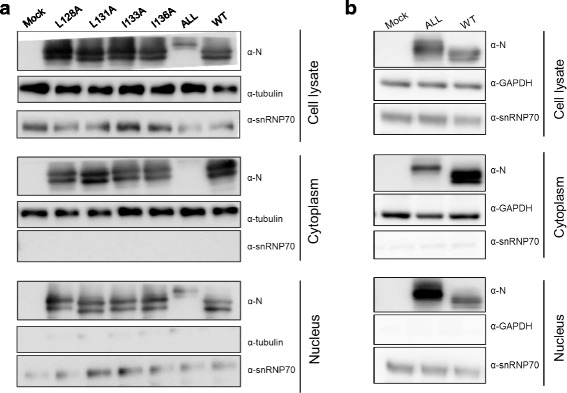
Subcellular localization of BoDV-N NES mutants. **a.** 293 T cells were transfected with pC-BoDV-MG-Gluc, pC-BoDV-N, pC-BoDV-L, and pCXN2-P. **b.﻿** 293 T cells were transfected with pC-BoDV-MG-Gluc, pC-BoDV-L, pCXN2-P and 87 ng of pC-BoDV-N (WT) or 2.1 μg of pC-BoDV-N-NES-A (ALL). **a** and **b.** At 48 h post-transfection, the cells were subjected to biochemical fractionation. BoDV-N and the fraction marker proteins (snRNP70 for the nucleus and β-tubulin or GAPDH for the cytoplasm) were detected by western blotting using anti-BoDV-N HN132 monoclonal antibody and anti-snRNP70 polyclonal, anti-β-tubulin, or anti-GAPDH monoclonal antibodies, respectively

Next we evaluated the subcellular localization of BoDV-N mutants using a biochemical fractionation assay [21]. 293 T cells in a 6-well plate were transfected with pC-BoDV-MG-Gluc, pC-BoDV-N, pC-BoDV-L, and pCXN2-P at the same ratio with minigenome assay using Lipofecatmine® 2000. At 48 h post-transfection, the cells were suspended in 500 μL HMKE buffer (20 mM HEPES [pH 7.2], 10 mM KCl, 5 mM MgCl_2_, 1 mM EDTA, 250 mM sucrose) containing 400 μg/mL of Digitonin (Nacalai Tesque, Kyoto, Japan). Cell suspensions were incubated for 20 min on ice and vortexed every 2 min, followed by centrifugation at 500×*g* for 10 min at 4 °C. The supernatant (cytoplasm fraction) and pellet (nuclear fraction) were assayed by SDS-PAGE using 5–20% gradient gels (ATTO, Tokyo, Japan). BoDV-N and the fraction marker proteins for the nucleus, snRNP70, and cytoplasm, as well as β-tubulin or glyceraldehyde-3-phosphate dehydrogenase (GAPDH), were detected by western blotting using anti-BoDV-N HN132 monoclonal antibody and anti-snRNP70 polyclonal antibody (Sigma-Aldrich, St. Louis, MO, USA), anti-β-tubulin monoclonal antibody (Sigma-Aldrich), and anti-GAPDH monoclonal antibody (Santa Cruz Biotechnology, Santa Cruz, CA, USA), respectively. As shown in Fig. 2a, the NES mutant with alanine-substitutions in all leucine and isoleucine residues was detected mainly in the nuclear fraction. In contrast, the other mutants showed no differences in intracellular localization compared to wild-type BoDV-N. To ensure that the protein expression levels of BoDV-N ALL mutant was comparable with wild-type, we performed the fractionation assay with the amount of transfection plasmid adjusted as follows: 87 ng for pC-BoDV-N (WT) and 2.1 μg for pC-BoDV-N-NES-A (ALL). As the result, the increased protein expression level of BoDV-N ALL mutant did not affect its cellular localization (Fig. 2b). Phyre2 analysis [22] predicted that the substitution of alanine into the NES increased the α-helical structure position from amino acids (aa) 130–149 to aa 128–149, causing differences in electrophoretic mobility among the NES mutants (Fig. 2a, b). These results indicate that the substitution mutants could not be exported to the cytoplasm, even in the presence of BoDV-P, and that nuclear export of N is important for efficient viral polymerase activity.

**Fig. 3:**
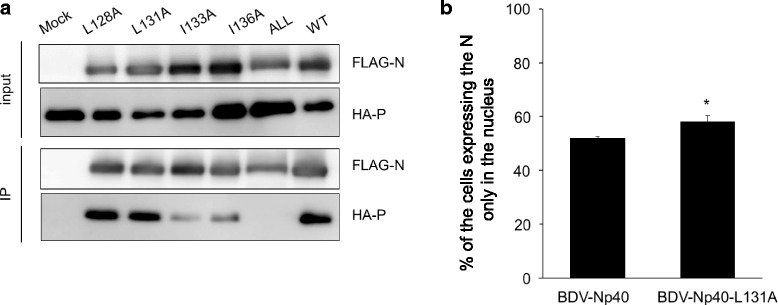
Interaction of BoDV-N NES mutants with BoDV-P. **a**. 293 T cells were transfected with pC-FLAG-BoDV-N, pC-BoDV-L, and pCNX2-HA-BoDV-P. At 48 h post-transfection, the cells were subjected to an IP assay using anti-FLAG M2 monoclonal antibody. The IP product was analyzed by western blotting using anti-FLAG M2 polyclonal and anti-HA monoclonal antibodies. **b**. Nuclear localization of BoDV-Np40 containing L131A mutation. OL cells were transfected with BoDV-Np40- or BoDV-Np40-L131-expression plasmids. At 48 h post-transfection, the cells were subjected to IFA using an anti-BoDV-N HN132 monoclonal antibody, and the percentages of cells expressing N only in the nucleus were calculated. A total of 150 cells were counted in 10 fields. The experiments were performed independently three times. The data were analyzed with Student’s *t*-test. Bars show mean ± standard deviation (SD) (*; *p* value <0.05)

Previous studies identified two P-binding regions in the N sequence (Fig. 1a) and demonstrated that higher expression of P inhibits nuclear export of N by masking the NES, leading to the regulation of viral replication in the nucleus [11, 16]. However, it has not been determined whether the N NES plays a direct role in the interaction with P. Thus, we assessed the interaction of NES mutants with BoDV-P. To evaluate the interaction between BoDV-N and BoDV-P, an immunoprecipitation (IP) assay was performed using N and P tagged with 3× FLAG tag and HA at the N-termini, respectively. 293 T cells were transfected with pC-FLAG-BoDV-N, pC-BoDV-L, and pCNX2-HA-BoDV-P. At 48 h post-transfection, the cells were harvested with lysis buffer (20 mM Tris-HCl, 150 mM NaCl, 1 mM EDTA, 1% Triton X-100, 1 U of protease inhibitor cOmplete [Sigma-Aldrich]). IP of the cell lysates was performed using anti-FLAG M2 antibody-conjugated Dynabeads Protein G (Thermo Fisher Scientific). The IP product was eluted with 1.5 μg of 3× FLAG peptide (Sigma-Aldrich) and subjected to western blotting. The results showed that although the single alanine-substitution mutants of BoDV-N NES were co-precipitated with BoDV-P, the mutant containing substitutions in all essential amino acids did not interact with P (Fig. 3a). The finding that all alanine-substitution mutants completely abolished the interaction with BoDV-P, despite the fact that the BoDV-N mutant retained a predicted major P-binding site at aa 51–100, indicated that the four residues in the BoDV-N NES are essential for the interaction with P. Considering that the amount of N in the nucleus is regulated by the interaction with P and is important for viral replication, it is very likely that during BoDV replication, the N NES has a dual function in nuclear export activity and P-binding.

To further evaluate the functional significance of the BoDV-N NES in viral replication, we next focused on mutant L131A, which showed the highest polymerase activity in the minigenome assay (Fig. 1c). To accurately evaluate the nuclear export activity of the BoDV-N L131A mutant, we generated a construct of BoDV-Np40, which did not express the p38 isoform when the second start codon of N was substituted with alanine (M14A). Based on the M14A mutant, we also constructed a BoDV-Np40 mutant with L131A, BoDV-Np40-L131A. The expression plasmids were transfected into oligodendroglioma (OL) cells. At 48 h post-transfection, the number of cells in which BoDV-N was localized to the nucleus or both the nucleus and cytoplasm were assessed by indirect fluorescent assay (IFA) using an anti-BoDV-N monoclonal antibody. As shown in Fig. 3b, the number of cells in which N was localized to the nucleus was slightly but significantly increased in BoDV-Np40-L131A compared to in BoDV-Np40.

**Fig. 4:**
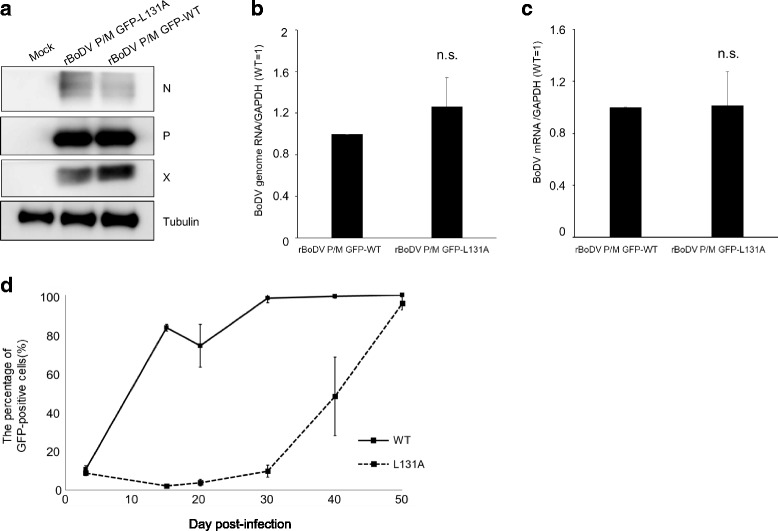
Characterization of rBoDV P/M-GFP-L131A. **a**. Expression levels of viral proteins in infected cells. Western blotting was performed with anti-BoDV-N HN132 monoclonal, anti-BoDV-P polyclonal, BoDV-X polyclonal, and anti-β-tubulin monoclonal (Sigma-Aldrich) antibodies. Expression levels of genomic RNA (**b**) and viral mRNA (**c**) in infected cells. Total RNA was extracted from infected cells and subjected to RT-qPCR. The experiments were performed independently three times. The data were analyzed with Student’s *t*-test. Bars show mean ± SD. **d**. Growth kinetics of rBoDV P/M-GFP-L131A. OL cells were infected with rBoDV P/M-GFP-L131A (L131A) or rBoDV P/M-GFP (WT) at a multiplicity of infection of 1. At days 3, 15, 20, 30, 40, and 50 post-infection, GFP-positive cells were counted using a Tali Image-Based Cytometer (Thermo Fisher Scientific). The experiments were performed independently three times. Bars show mean ± SD

The results shown above suggest that the L131A mutant mildly impairs the efficiency of nucleocytoplasmic shuttling of N, resulting in enhanced polymerase activity in the minigenome assay. To confirm this, we rescued rBoDV P/M-GFP, which contains the GFP sequence between the P and M genes, and rBoDV P/M-GFP-L131A, which contains an L131A mutation in the N gene, by reverse genetics as previously described [23]. A total of 5.0 × 10^5^ 293 T cells were transfected with 2 μg of pC-BoDV P/M-GFP with L131A in N, 0.25 μg of pC-BoDV-L, 0.025 μg of pCXN2-P, and 0.25 μg of pC-BoDV-N-L131A. In parallel, we rescued rBoDV P/M-GFP-WT. At 72 h post-transfection, 293 T cells were co-cultured with 4 × 10^5^ of puromycin-resistant Vero cells and passaged every 3 days with 1 μg/mL of puromycin to remove transfected 293 T cells. After several passages, we confirmed the establishment of persistently infected Vero cells based on rBoDV and expression of viral proteins in both cells at a comparable level (Fig. 4a). Western blotting was performed with SDS-PAGE using 5–20% gradient gels, and BoDV proteins were detected with anti-BoDV-N HN132 monoclonal, anti-BoDV-P polyclonal, and BoDV-X polyclonal antibodies.

To evaluate whether the N L131A mutant enhanced polymerase activity in the replication-competent virus, transcription and replication activity in rBoDV-infected cells were quantified by real-time qPCR. The extracted RNA from the viral-infected cells was reverse-transcribed using the Verso cDNA Synthesis Kit (Thermo Fisher Scientific) using an oligo dT primer or viral gene-specific primer (5′ -GTT GCG TTA ACA ACA AAC CAA TCA T-3′). The cDNA was subjected to real-time PCR using THUNDERBIRD™ SYBR® qPCR MIX (Toyobo, Osaka, Japan). Real-time qPCR was performed using primers (5′ -ATG CAT TGA CCC AAC CGG TA-3′ and 5′ -ATC ATT CGA TAG CTG CTC CCT TC-3′) to amplify viral mRNA and genomic RNA. Primers (5′-ATC TTC TTT TGC GTC GCC AG-3′ and 5′-ACG ACC AAA TCC GTT GAC TCC-3′) were used to amplify GAPDH mRNA. Real-time qPCR showed that the expression levels of the viral mRNA and genomic RNA in rBoDV P/M-GFP-L131A-infected cells were similar to that in the rBoDV P/M-GFP-WT-infected cells (Fig. 4b, c), suggesting that the slight impairment in nucleocytoplasmic shuttling of N can be overcome by the interaction with other viral proteins, such as X and L, in infected cells.

To further evaluate the effect of alanine-substitution of leucine at position 131 in the NES on virus growth, the growth kinetics of rBoDV P/M-GFP-L131A were assessed. OL cells were infected with rBoDV P/M-GFP-L131A or rBoDV P/M-GFP-WT at a multiplicity of infection of 1, and the percentage of GFP-positive cells was counted using a Tali Image-Based Cytometer (Thermo Fisher Scientific) on days 3, 15, 20, 30, 40, and 50 post-infection. Although the growth of rBoDV P/M-GFP-L131A was slower than that of rBoDV P/M-GFP-WT, the infection rate of both viruses reached 100% by day 50 post-infection (Fig. 4d), suggesting that alanine-substitution of leucine at position 131 in the NES slightly impaired virus growth.

We showed that alanine-substitution of the leucine at position 131 in the NES of BoDV-N increased viral polymerase activity in the minigenome assay, but did not play a key role in the replication cycle of rBoDV. Similarly, it was previously reported that one amino acid mutation in N of human parainfluenza 1 virus type 2 (hPIV2), which belongs to the same order as BoDV—*Mononegavirales*, markedly enhanced polymerase activity in the minigenome assay, but the recombinant hPIV2 containing the mutation could not be rescued [24]. This suggests that although the minigenome assay is often used because of its convenience, polymerase activity of the actual virus is not accurately reflected by it.

We demonstrated here that the leucine and isoleucine residues in the BoDV-N NES have a dual function in nuclear export activity and P-binding, which are essential for BoDV replication. Our findings increase the understanding of the mechanism of persistent infection of BoDV in the nucleus.

## Abbreviations

aa: Amino acid
BoDV: Borna disease virus, NES: nuclear export signal
GAPDH: Glyceraldehyde-3-phosphate dehydrogenase
Gluc: Gaussia luciferase
hPIV2: human parainfluenza 1 virus type 2
IP: Immunoprecipitation
NLS: Nuclear localization signal
OL: Oligodendroglioma
vRNA: viral RNA
vRNP: ribonucleoprotein of BoDV
